# Depression among Medical Students of a Medical College: A Descriptive Cross-sectional Study

**DOI:** 10.31729/jnma.7869

**Published:** 2023-03-31

**Authors:** Bharat Khatri, Baidh Gupta, Santosh B.K., Rinku Gautam, Atit Tiwari, Anushka Khanal, Bebika Subedi, Dipen Dhakal, Rabina Adhikari

**Affiliations:** 1 B.P. Koirala Institute of Health Sciences, Dharan, Sunsari, Nepal; 2 Department of Psychiatry, B. P. Koirala Institute of Health Sciences, Dharan, Sunsari, Nepal; 3 Nepal Medical College and Teaching Hospital, Jorpati, Kathmandu, Nepal; 4 Sanjeevani College of Medical Sciences, Butwal, Rupandehi, Nepal

**Keywords:** *depression*, *medical students*, *mental health*

## Abstract

**Introduction:**

Medical students encounter multiple psychological changes in the transformation from young insecure students to efficient physicians. They have to balance the personal, social and academic dimentions in a busy schedule. This study aimed to find out the prevalence of depression among medical students of a medical college.

**Methods:**

A descriptive cross-sectional study was conducted among medical students of a medical college from 2 May 2017 to 16 October 2017 after taking ethical approval from the Departmental Research Unit (Reference number: Psy/73/078/079). Students participated voluntarily in the study from first to fourth year and written informed consent was taken. Depression, Anxiety and Stress Scale-42 scale was filled by the students taking their own time and privacy to assess their depression, anxiety and stress. Convenience sampling was done. Point estimate and 95% Confidence Interval were calculated.

**Results:**

Among 302 medical students, 86 (28.47%) (23.38-33.56, 95% Confidence Interval) had depression. A total of 31 (36.04%) had mild, 31 (36.04%) had moderate, 12 (13.95%) had severe and 12 (13.95%) had extremely severe depression. Among them 55 (63.95%) were males and 31 (36.04%) were females.

**Conclusions:**

The prevalence of depression among medical students was similar to the other studies conducted in similar settings. Studies concerning the subjective well-being of medical students should be continued and strategic plans and programs should be conducted to help the students manage their stress and depressive symptoms right from the time they join the medical school and continued till they finish the course.

## INTRODUCTION

Medical students, destined to become proficient doctors, expected to have complete information to absorb, haunted with self-doubt over their abilities, suffer from burnout and a significant number face depression.^1^ In Brazil 32.8% had depression.^2^ Studies showed that 70% of medical students have depression in Pakistan and almost half of the medical students have depression in India.^3^ In Nepal the prevalence of depression was found to be 29.9% in medical students.^4,5^

The prevalence of depression among medical students in Nepal could provide information about ongoing depression which can be a baseline for future research and planning any preventive strategies.

Therefore, the aim of this study was to find out the prevalence of depression among the medical students of a medical college.

## METHODS

A descriptive cross-sectional study was conducted among medical students of B.P. Koirala Institute of Health Sciences (BPKIHS) from 2 May 2017 to 16 October 2017 after receiving ethical approval from the Departmental Research Unit (DRU), BPKIHS (Reference number: Psy/73/078/079). All MBBS students of BPKIHS from the first to the fourth year who provided informed written were included. The students who did not give informed written consent and those who were absent during the study period were excluded. Data was collected using convenience sampling. The sample size was calculated using the following formula:


$$\begin{array}{ll} \text{n} & ={\text{Z}}^{2}\times \frac{\text{p}\times \text{q}}{{\text{e}}^{\text{2}}}\\ & =1.64^{2}\times \frac{0.299\times 0.701}{0.06^{2}}\\ & =227 \end{array}$$

Where,

n = minimum required sample size
Z = 1.96 at 95% Confidence Interval (CI)
p = prevalence of depression among the medical students, 29.9%^4^
q = 1-p
e = margin of error, 6%

The minimum required sample size was 224. After adding a 20% non-response rate, the sample size of 280 was calculated. However, 302 students were taken for the study. All MBBS students were informed about the purpose of the study and explained the general instructions. Informed written consent was taken prior to the study. The students were allowed to respond in their own time and privacy. The participation was entirely voluntary. Then they were given the questionnaires containing semi-structured sociodemographic profiles and the Depression, Anxiety and Stress Scale (DASS-42).^6^

Data were entered and analysed in IBM SPSS Statistics 11. 5. Point estimate and 95% CI were calculated.

## RESULTS

**Figure 1:**
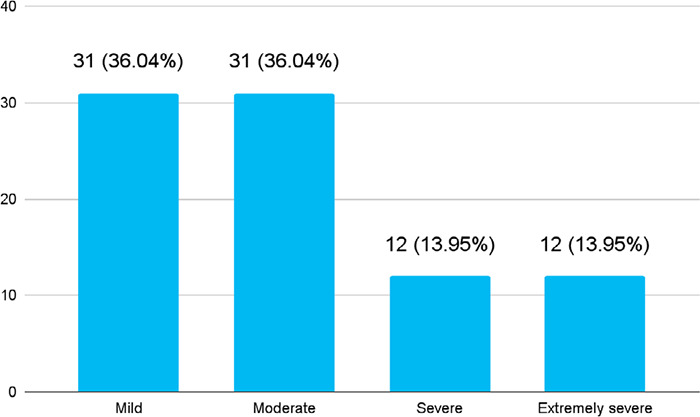
The severity of depression among MBBS students (n= 86).

Among 302 medical students, depression was seen in 86 (28.47%) (23.38-33.56, 95% CI). Among them, 31 (36.04%) had mild depression, 31 (36.04%) had moderate depression, 12 (13.95%) had severe depression and the remaining 12 (13.95%) had extremely severe depression (Figure 1).

Males were 55 (63.95%) and females were 31 (36.04%) Past history of depression was found in 9 (10.46%) students. A total of 19 (41.30%) and 8 (61.54%) of the medical students whose father's and mother's occupation was doctor were depressed. Similarly, 30 (29.13%) of the depressed students had a family monthly income of less than Rs. 50000. History of substance abuse was in 15 (42.86%) students and a history of mental illness in 9 (69.23%) (Table 1).

**Table 1:** Characteristics of the students with depression (n= 86).

Variables	Depression n (%)
**Sex**	
Male	55 (63.95)
Female	31 (36.04)
**Father's occupation**	
Business	20 (25.97)
Farmer	5 (29.41)
Doctor	19 (41.30)
Engineer	6 (33.33)
Teacher	10 (20.83)
Others	26 (30.23)
**Mother's occupation**	
Housewife	47 (24.48)
Farmer	3 (3.48)
Doctor	8 (61.54)
Teacher	18 (34.62)
Others	10 (11.62)
**Family monthly income (in Nepalese rupees)**	
<50,000	41 (47.67)
50,000 to 100,000	28 (32.55)
>100,000	17 (19.76)
**Substance use**	15 (17.44)
**Past history of mental illness**	9 (10.46)
**Family history of mental illness**	14 (16.27)

## DISCUSSION

In our study, among undergraduate medical students, 28.47% had depression. The findings were consistent with the studies done in Brazil (32.8%),^2^ and Nepal (29.9%),^4^ but lesser than in India where depression among MBBS students was 51.3%.^7^ However a study done among undergraduates in Sweden showed the prevalence to be 12.7% which was lower than in our study.^8^ Also, the categorical prevalence of mild 31 (36.04%), moderate 31 (36.04%) and severe depression 12 (13.95%) among the MBBS students in our study is consistent with the studies conducted elsewhere.^9,10^ However, the prevalence of extremely severe depression 12 (13.95%) in our study was higher than in India (1.32%).^9^ The differences in an academic environment, the availability of entertainment, sports and club activities, the policy regarding mental health and the student's own genetic and socio-demographic variables may be responsible for the variation in the prevalence of depression among the students in different medical colleges.

In our study, male students 55 (64.00%) had a higher prevalence of depression when compared to female students 31 (36.00%). This finding is in contrast to findings from other similar studies.^7-9^ The variation may be due to the gender-based disproportionate enrolment of students in our medical college. Additionally, females reported being involved in more club activities, social gatherings and tours; which might have helped them to cope with stress and maintain sound mental health. However, a different study among Turkish undergraduate students has shown no significant differences between the mean depression scores of male and female students.^11^

In our study, 19 (22.09%) of the medical students' fathers and 8 (9.30%) of the students' mothers were doctors. The study conducted in Brazil showed students having their parents as doctors had higher rates of depression.^2^ Majority 41 (47.67%) of the students having depressive symptoms had poor socioeconomic conditions. The finding is consistent with other similar studies.^11-12^

In our study, 15 (17.46%) of the students had a history of substance abuse. The finding is similar to the research conducted among north Indian medical students (20.43%).^13^ However; it was lower than the Nigerian medical students (28%).^14^ The common substances of abuse were alcohol and cigarettes. Likewise, 9 (10.46%) of the students having depressive symptoms reported having a past history of depression whereas, 14 (16.27%) of them reported having a family history of depression. Different studies also support the genetic basis of depression.^15^'^17^

Hurryingly filling up questionnaires by students may have resulted in an incomplete grasp of questions leading to a different result from the existing conditions. The diagnosis was only done by self administered DASS-42 and no observation was done by a consultant/psychiatrist leading to inadequate authenticity of the result. All were hostellers and this couldnot reflect the scenario of students living in the home. Only students from single institution were involved and can not be generalised as it does not include all medical students from other colleges of Nepal.

## CONCLUSIONS

The prevalence of depression among MBBS students was similar to the other studies conducted in similar settings. Results indicate a substantial number of students are suffering from depression. Beside from improving medical education to more humanistic and student-centered, policy makers and other concerned stakeholders should plan to identify and help them to cope with the depression begining from the first year of medical school.
